# Parallel mutual information estimation for inferring gene regulatory networks on GPUs

**DOI:** 10.1186/1756-0500-4-189

**Published:** 2011-06-15

**Authors:** Haixiang Shi, Bertil Schmidt, Weiguo Liu, Wolfgang Müller-Wittig

**Affiliations:** 1 School of Computer Engineering, Nanyang Technological University, Singapore

## Abstract

**Background:**

Mutual information is a measure of similarity between two variables. It has been widely used in various application domains including computational biology, machine learning, statistics, image processing, and financial computing. Previously used simple histogram based mutual information estimators lack the precision in quality compared to kernel based methods. The recently introduced B-spline function based mutual information estimation method is competitive to the kernel based methods in terms of quality but at a lower computational complexity.

**Results:**

We present a new approach to accelerate the B-spline function based mutual information estimation algorithm with commodity graphics hardware. To derive an efficient mapping onto this type of architecture, we have used the Compute Unified Device Architecture (CUDA) programming model to design and implement a new parallel algorithm. Our implementation, called CUDA-MI, can achieve speedups of up to 82 using double precision on a single GPU compared to a multi-threaded implementation on a quad-core CPU for large microarray datasets. We have used the results obtained by CUDA-MI to infer gene regulatory networks (GRNs) from microarray data. The comparisons to existing methods including ARACNE and TINGe show that CUDA-MI produces GRNs of higher quality in less time.

**Conclusions:**

CUDA-MI is publicly available open-source software, written in CUDA and C++ programming languages. It obtains significant speedup over sequential multi-threaded implementation by fully exploiting the compute capability of commonly used CUDA-enabled low-cost GPUs.

## Background

Mutual information (MI) is used to measure the mutual dependence between two random variables in information theory. As an information theoretic approach, it has been used in various areas including physics [1], image processing [2,3], speech recognition [4], and bioinformatics [5,6]. An advantage of MI compared to many other similarity measures (such as Pearson correlation), is its capability to detect non-linear correlations between the two variables [7]. In [1], a recursive method for calculating MI is presented and used on dynamical systems and chaotic data. An overview about MI used in medical imaging applications is presented in [2]. An MI application to find features for audio-visual speech recognition tasks is proposed in [4]. Zhou et al. [5] used MI for gene clustering to determine gene regulations. In [6], MI is used to measure nonlinear relationships between the expressions of two genes. Because of its inherent algorithmic complexity, the reverse engineering or inference of gene regulatory networks (GRNs) from gene-expression profile (microarray) data remains a big challenge in system biology [7-9]. Approaches such as relevance networks [10], information-theoretic methods [11], and Bayesian networks [12,13] have been used to infer GRNs. However, due to their high computational complexities, these methods are very time-consuming and cannot be used to process large datasets. Daub et al. [7] proposed a B-spline function based MI estimation algorithm which can achieve accuracy comparable to kernel-based MI approaches. Since the B-spline based approach has a lower computational complexity, it is widely used in practice. However, it is still highly time-consuming for big gene expression datasets. Since availability and size of such datasets is growing rapidly, finding fast solutions is of high importance to research in this area.

In this paper, we present a new approach to accelerate B-spline function based MI estimation using the CUDA programming model. We take advantage of shared memory for fast I/O operations to gain efficiency. We further use double precision floating point arithmetic as well as an efficient partitioning scheme to overcome the GPU device memory limitation for big datasets. We evaluate our implementation for a number of microarray datasets. It achieves speedups up to 82 on an Nvidia Tesla C2050 GPU compared to the publicly available multi-threaded implementation by Daub et al. [7] running on an Intel i7 quad-core CPU. We use our MI results to infer GRNs by replacing the time-consuming MI calculation in ARACNE [14]. The results show that the runtime needed for inferring GRNs is much shorter and quality of the resulting network is better by using our MI values compared to the original ARACNE.

The paper presented by Wilson et al. [15] is similar to the approach presented in this paper since it also uses CUDA-enabled GPUs to accelerate B-spline based MI estimation. However, it only implements the weighting matrix computation on the GPU. Weighting matrix calculation is only one step in the B-spline based MI estimation algorithm. The remaining steps are performed on the CPU, thus limits the possible speedup. The solution presented in this paper overcomes this limitation by accelerating all steps of the B-spline based MI estimation using multiple CUDA kernels. Our experiments therefore show significant higher speedups than the ones reported in [15].

### B-spline Based Mutual Information Estimator

#### Definition of Mutual Information

Mutual information (MI) is used as a quantity to measure the dependence of two discrete random variables. For a random variable *X *with values over a finite set of *M *possible values (or states) {*x*_1_, *x*_2_, ..., *x_M_*}, the Shannon entropy *H*(*X*) is defined as follows:(1)

where *p*(*x_i_*) is the probability of the value *x_i_*. The Shannon entropy is a measure of the uncertainty of *X*. If the outcome of a measurement of *X *is completely determined, then *p*(*x_i_*) = 1 and the entropy will be 0. The joint entropy *H *(*X*, *Y *) of two discrete variables (*X *and *Y *) consisting of states {*x*_1_, ..., *x_M_*} and {*y*_1_, ... *y_M _*} can be defined by the following equation:(2)

where *p*(*x_i_*, *y_j_*) is the joint probability between two states *x_i _*and *y_j_*. The joint entropy denotes the quantity of entropy contained in a joint system of two variables. The MI for *X *and *Y *can then be defined by:(3)

From Eqn. (3), we can see that the MI is zero when *X *and *Y *are independent

#### Estimating Mutual Information for Continuous Data

MI can be easily estimated for discrete data. In order to estimate MI for continuous data (or measurements) which are supplied by many practical applications, a common method is to divide the continuous values into *R *discrete bins {*a*_1_, ..., *a_R_*}. Each bin may contain several data points. Assume the continuous dataset consists of *M *measurements {*x*_1_, ..., *x_M_*}. The indicator function *θ_j _*is used to count the number of measurements within each bin *a_j _*(*j *= 1, ..., *R*) [7]. The probability for each bin then equals to the number of measurements within the bin divided by the total number of measurements (see Eqn. (4)).(4)

The indicator function *θ_j _*is a binary function defined as:(5)

The joint probability *p*'(*a_j_*, *b_k_*) for two random variables with measurements {*x*_1_, ..., *x_M_*} and {*y*_1_, ..., *y_M _*} and two given bins *a_j _*and *b_k_*, can be calculated as follows:(6)

After estimating the probabilities using Eqns. (4) and (6), we can compute MI using Eqns. (1) to (3). In the simple binning method mentioned above, each continuous value is assigned to exactly one bin. Values near to the border of a bin can be shifted to neighboring bins by small fluctuations. Thus, the MI result is strongly affected by noise. In order to overcome this shortcoming of the simple binning method, Daub et al. [7] introduced the B-spline approach. In Daub's approach, each measurement can be assigned to multiple bins with weights given by B-spline functions.

#### B-spline Functions

We use the same knot vector in the B-spline function as in [7]. A knot vector *t_i _*with *R *bins and the spline order *k *is defined in Eqn. (7). The spline order *k *should meet the condition *k *∈ {1, ..., *R *- 1}(7)

The degree of the polynomial functions is determined by the spline order *k*. The B-spline function is defined as follows.(8)

where *i *∈ [1, *R*] and *z *∈ [0, *R *- *k *+ 1] is the domain interval of the B-spline function. Note that continuous values should be normalized to fit into the domain interval. Assume we have *M *continuous data points in the dataset, *x*_1_, ..., *x_M _*, the normalization procedure is as follows.

1. Find the minimum and maximum value *x_min _*and *x_max _*among all *M *data points.

2. Compute the normalized value *z_i_*, (*i *= 1, ..., *M*) for the continuous value using(9)

where *R *is the number of bins used in the B-spline algorithm and *k *is the spline order of the B-spline function.

#### Sequential MI estimator

Our parallel CUDA algorithm (described in Methods) extensively uses the concept of the weighting matrix of each given random variable, which is defined as follows.

**Definition **Weighting Matrix (WM): Consider a random variable *X *= {*x*_1_, *x*_2_, ..., *x_M_*}, *R *bins {*a*_1_, ..., *a_R_*}, and the B-spline function order *k*. The weighting matrix for *X *is an *M *× *R *matrix denoted as *W *(*X*), where *W *(*X*)*_i_*_,_*_j _*contains weighting coefficient of value *x_i _*in bin *a_j_*; i.e. *W *(*X*)*_i_*_,_*_j _*= *B_j_*_,_*_k _*(*z_i_*) where *z_i _*is the B-spline domain normalized value of *x_i _*for each 1 ≤ *i *≤ *M *and 1 ≤ *j *≤ *R*.

Based on Eqns. (1) to (8), we can now outline the sequential B-spline based MI estimation algorithm. It consists of two parts: *WM*(*X*, *R*, *k*) and *Single*_*MI *(*X*, *Y*, *R*, *k*), which are described in Algorithm 1 and Algorithm 2.

**Algorithm 1**: *W M*(*X*, *R*, *k*)

**   Input**: Random variable *X *= {*x*_1_, ..., *x_M _*}, number of bins *R*, B-Spline order *k*

   **Output**: *W *(*X*)

   **foreach ***i*, 1 ≤ *i *≤ *M ***do**

      Calculate the normalized variable *z_i_*, (*i *= 1, ..., *M*) using Eqn. (9);

      **foreach ***j*, 1 ≤ *j *≤ *R ***do**

         Calculate the weighting coefficient *B_j_*_,_*_k _*(*z_i_*) using Eqn. (7) and (8) with the normalized value

         *z_i _*;

      **end**

   **end**

   Output *W *(*X*)*_i_*_,_*_j _*= *B_j_*_,_*_k _*(*z_i_*)

**Algorithm 2**: *Single_M I *(*X*, *Y*, *R*, *k*)

   **Input**: Random variable measurements *X *= {*x*_1_, ..., *x_M_*} and *Y *= {*y*_1_, ...,*y_M_*}, number of bins *R*, B-Spline order *k*

   **Output**: *M I *(*X*, *Y *)

   Call *W M *(*X*, *R*, *k*) to get *W *(*X*);

   Call *W M *(*Y*, *R*, *k*) to get *W *(*Y*);

   **foreach ***j*, 1 ≤ *j *≤ *R ***do**

      Calculate the probability of each bin for *X *and *Y *;

      

      

   **end**

   Calculate the self entropy *H *(*X*) and *H *(*Y*);

   

   

   **foreach ***k*, 1 ≤ *k *≤ *R ***do**

      **foreach ***l*, 1 ≤ *l *≤ *R ***do**

         Calculate joint probabilities;

         

      **end**

   **end**

   Calculate the joint entropy;

   

   Calculate the mutual information using Eqn. (3);

The *Single_MI *algorithm shows how the MI for one pair of random variables is calculated. Practical applications of MI usually have a large number of random variables as input, where the mutual information of each pair of variables needs to be computed. For example, in this paper we are interested in the analysis of gene expression data, where the input consists of *N *genes Ω = {*X*_1_, ..., *X_N_*}, where *N *is typically a few thousands. For each gene *X_i _*we have *M *gene expression measurements; i.e. *X_i _*= {*x_i_*_1_, ..., *x_iM _*}. We then want to calculate *M I *(*X_i_*, *X_j_*) for all 1≤ *i *≤ *N *- 1, *i *<*j *≤ *N*. The resulting matrix of pairwise MI values can be used as input to a subsequent clustering algorithm. The algorithm to calculate all pairwise MI values is outlined in Algorithm 3.

**Algorithm 3**: *Pairwise*_*MI *(Ω, *R*, *k*)

   **Input**: *N *Random Variables Ω = {*X*_1_, ..., *X_N_*} consisting of *M *measurements each; i.e., *X_i _*= {*x_i_*_1_, ..., *x_iM _*} for all 1 ≤ *i *≤ *N*; number of bins: *R*; B-Spline order: *k*.

   **Output**: *M I *(*X_i_*, *X_j_*) for all 1 ≤ *i *≤ *N ***- **1, *i *<*j *≤ *N*

   **foreach ***i*, 1 ≤ *i *≤ *N ***do**

      *W M *(*X_i_*, *R*, *k*);

   **end**

   **foreach ***i*, 1 ≤ *i *≤ *N ***- **1 **do**

      **foreach ***j*, *i *≤ *j *≤ *N ***do**

         *Call Single *_*MI *(*X_i_*, *X_j_*, *R*, *k*) to calculate MI for this gene pair using Algorithm 2;

      **end**

   **end**

### Complexity Analysis

The most time consuming step of Algorithm 1 is the inner for-loop. Assuming that the evaluation of a B-spline function call takes *O*(*k*) time, the time complexity of this step is *O*(*M *× *R *× *k*). We further need *O *(*M *× *R*) space to store the output weighting matrix. Since *W M *(*X_i_*, *R*, *k*) is called *N *times in Algorithm 3, this leads to a time complexity of *O *(*N *× *M *× *R *× *k*) and space complexity of *O *(*N *× *M *× *R*) for the first for-loop. The nested for-loop of Algorithm 3 calls Algorithm 2 *O*(*N*^2^) times. The time consuming part of Algorithm 2 is determined by the nested for-loop which has time complexity *O *(*M *× *R*^2^). Thus, the overall time complexity of Algorithm 3 is *O *(*N*^2 ^× *M *× *R*^2^). Note that *k *and *R *are usually significantly smaller than *N *and *M*.

### CUDA Programming Model

As an extension of C/C++, CUDA (Compute Unified Device Architecture) is used to write scalable multithreaded programs for CUDA-enabled GPUs [16]. CUDA programs can be executed on GPUs with NVIDIA's Tesla unified computing architecture. Examples of CUDA enabled GPUs ranging from GeForce 8/9/200, Tesla 800/1000, C1060, C2050, to Quadro FX 3000/4000/5000 series.

CUDA programs contain a sequential part, called the kernel program. The kernel is written in conventional scalar C-code. It represents the operations to be performed by a single thread and is invoked as a set of concurrently executing threads. These threads are organized in a hierarchy consisting of so-called thread blocks and grids. A thread block is a set of concurrent threads and a grid is a set of independent thread blocks. Each thread has an associated unique ID. Similar to MPI processes, CUDA provides each thread access to its unique ID through corresponding variables. The total size of a grid (dimGrid) and a thread block (dimBlock) is explicitly specified in the kernel function-call:

*Kernel *<<<*dimGrid*, *dimBlock *>>>*(parameter list)*;

The hierarchical organization into blocks and grids has implications for thread communication and synchronization. Although threads located in different blocks cannot communicate or synchronize directly, threads within a thread block can communicate through a per-block shared memory (PBSM) and may synchronize using barriers. The Tesla architecture supports CUDA applications using a scalable processor array. The array consists of a number of streaming multiprocessors (SM). In the latest Fermi architecture [17], each SM contains 32 scalar streaming processor (SP) cores, which share a PBSM of size up to 48 KB. All threads of a thread block are executed concurrently on a single SM. The SM executes threads in small groups of 32, called warps, in single-instruction multiple-thread (SIMT) fashion. Thus, parallel performance is generally penalized by data-dependent conditional branches and improved if all threads in a warp follow the same execution path. There are two types of parallelism supported by CUDA. First, a large number of threads can run in parallel independently. We call this type of parallelism the coarse-grained. Second, multiple threads within each thread blocks can co-operate on some memory spaces (such as the shared memory) simultaneously. For instance, shared memory I/O request made of n addresses can be serviced in a single clock cycle at the same time. Threads in the same thread block can cooperate together by efficiently sharing data and synchronizing their execution to coordinate memory access with other threads. We call this type of parallelism fine-grained.

Previous work on using CUDA for computational biology focused on sequence alignment [18-21], and molecular dynamics simulations [22]. In this paper we present the parallel B-spline function based MI estimation which can help to infer GRNs using CUDA. So far, parallel MI estimation using B-splines has been limited to multi-threading on multi-core CPUs [7] and MPI on distributed memory clusters [8,23]. The results presented in this paper indicate that the CUDA approach can provide higher performance at reasonable hardware cost.

## Methods

### Parallel MI Estimation using CUDA

**Algorithm 4**: CUDA-based MI estimation algorithm

**Input**: *N *genes, each with *M *experiments.

**Output**: Pairwise MI values.

/*Host programs executed on CPU*/

Initialize parameters controlling MI estimation;

Load gene expression data into GPU device memory and launch the kernels;

/*Kernel program executed on GPU*/;

Compute the WM for each gene (Kernel 1);

Check the data integrity of the input data (Kernel 2);

Compute the self entropy for each gene (Kernel 3);

Compute the joint entropy and MI value for each gene pair (Kernel 4);

/*Host programs executed on CPU*/

**Figure 1:**
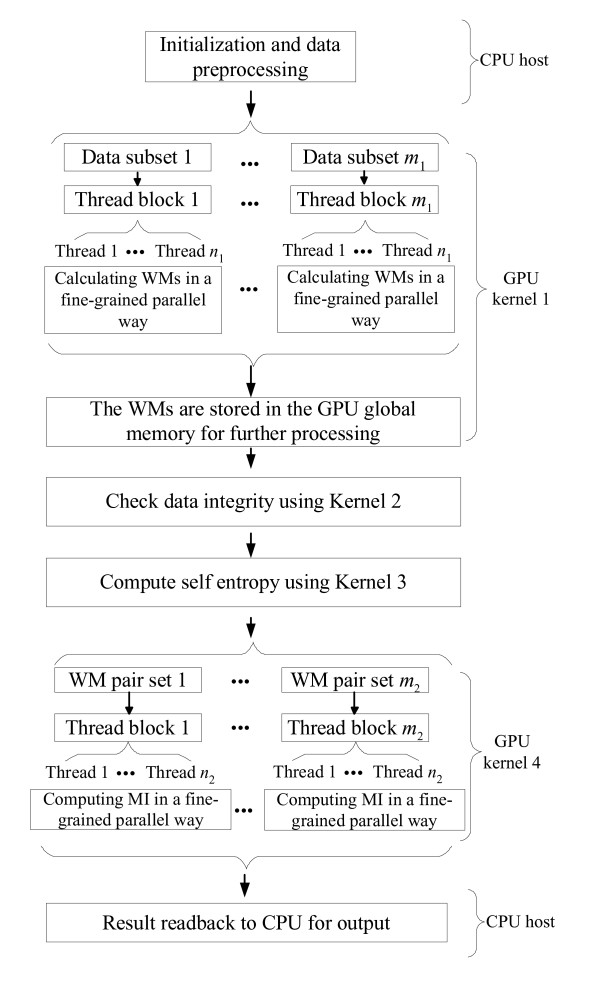
**The algorithm framework for CUDA-MI**.

Read back results to CPU and output;

Algorithm 4 shows the pseudocode of our CUDA-based MI estimation algorithm. There are four CUDA kernel programs in our algorithm. In Kernel 1 the gene expression data is divided evenly into subsets according to the total number of thread blocks. All thread blocks then work in parallel to compute the probabilities for the local gene subset using the Algorithm 1. The probabilities of each gene are stored in the WMs. In Kernel 1, threads in the same thread block process each WM in a fine-grained parallel fashion. Before computing the self entropy for each gene using Kernel 3, we use Kernel 2 to check the integrity of the gene expression data first. The execution of Kernel 2 is necessary if there are blank values (caused by missing experiments) in the gene expression data. In Kernel 4, WM pairs are distributed evenly to all thread blocks. Threads in the same thread block then work in a fine-grained way to compute the MI value for each gene pair. Figure 1 shows our algorithm framework for CUDA-MI.

### Parallel Computation of WM

**Figure 2:**
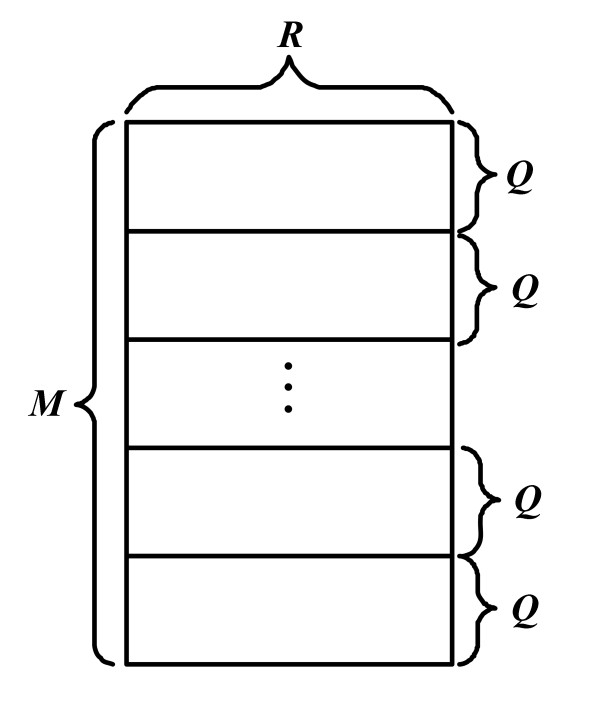
**Assuming there are R bins and M rows in a WM**. We partition the WM into small batches so that the size of each batch is *Q *× *R*.

**Figure 3:**
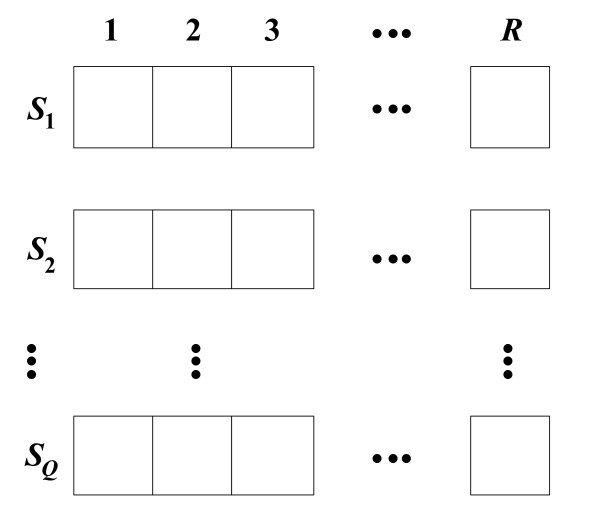
**There are *R *bins and *Q *rows in a WM batch**. *S*_1_, *S*_2_, ..., *S_Q _*are shared memory arrays to store each row of the batch. The size of each shared memory array is *R *values (the number of bins).

Kernel 1 is used to compute WMs from gene expression data. We take advantage of the inherent parallelism of WM calculation and design parallel algorithms using the fine-grained method. In our algorithm, the gene expression data is first divided evenly into subsets according to the total number of thread blocks. Each thread block then computes WMs for the allocated subsets of gene expression data. Since all elements in the same row of the WM can be computed independently, the WM can be computed row by row in parallel. In order to speed up the I/O operations, the rows are stored in high-performance shared memory arrays. The size of each shared memory array is *R *values, which equals the number of bins. In the kernel program there are *R *threads working in the ne-grained concurrent way to calculate each row of the WM. Assuming there are *M *rows in a WM, we partition the WM into small sized batches so that each batch can be mapped into the shared memory (see Figure 2). In Figure 2, the size of each batch is *Q *× *R*. The WM can then be computed batch by batch. Figure 3 shows that totally *Q *× *R *shared memory space is required to calculate each batch.

In practice, there are *R *+ *k *- 1 threads working in Kernel 1 in the fine-grained concurrent way to calculate each row of the WM. Thus there are totally *Q *× (*R *+ *k *- 1) threads to compute the WM for each gene. It should be noted that the computation of the WM for each gene may span into different thread blocks because the maximum threads for each GPU block is limited and the size of the WM is varying with the size of the experiment *M*, the bin number *R *and the spline order *k*.

**Figure 4:**
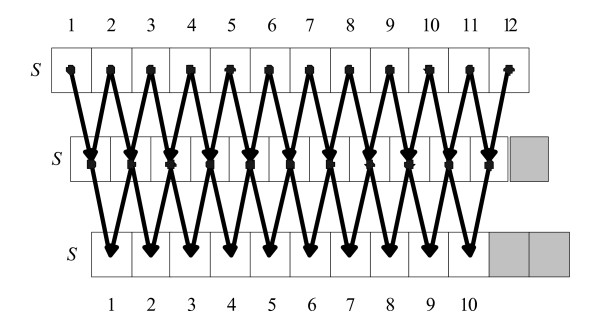
**Illustration of unwinding the recursive Eqn. (8) into *k *dependent steps using *k *= 3 and *R *= 10 for computing one row of a WM**. *S *is the shared memory array. In each step, 12 threads work together to compute the values of S. After 3 steps, the first 10 values in *S *is the final result.

The reason for using *R *+ *k *- 1 threads instead of *R *threads for computing each row of WM is because the B-spline function in Eqn. (8) is recursively defined. In order to compute each row of the WM efficiently, we unwind the recursion relationships in Eqn. (8) into *k *dependent steps. In each step, *R *+ *k *- 1 threads work in the fine-grained concurrent way to calculate the values of the shared memory array. In the first step, the shared memory array is initialized using Eqn. (8). In the subsequent step, the calculation of each cell on the shared memory array follows the dependency relationship in Figure 4. After the last step is done, the first *R *values of the shared memory array are the final results for the current row calculation. Figure 4 illustrates this method for the parameters *k *= 3 and *R *= 10.

After all WMs are computed, Kernel 2 will be invoked to check the data integrity. Kernel 3 then follows the first for-loop of Algorithm 2 to compute the self entropy for each gene. WMs and the self entropy calculated in Kernel 1 and 3 will be passed to Kernel 4 to compute the MI values for all gene pairs.

### Parallel Computation of MI

**Figure 5:**
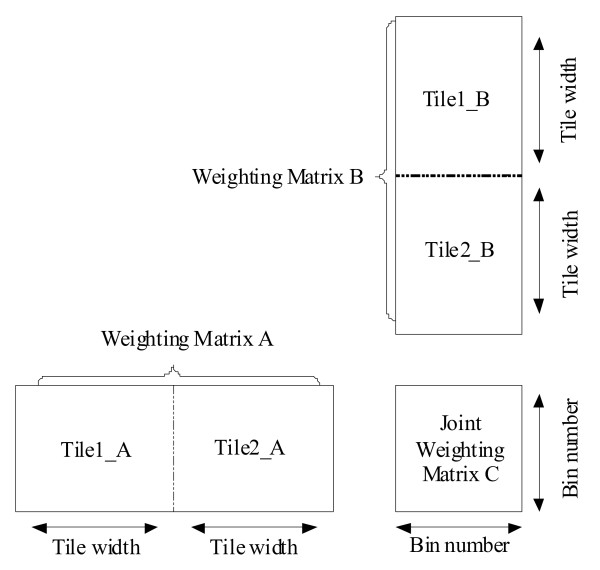
**The tiled method to carry out matrix multiplication operations between two WMs**.

According to Algorithm 3, two steps are involved in Kernel 4. Firstly, the joint entropy should be calculated using Eqn. (2) and (6). Secondly, we need to compute the MI values using Eqn (3). Profiling of these two steps for different datasets reveals that more than 99% of the overall runtime is spent on the first step. In order to calculate the joint entropy for a pair of genes, we need to perform matrix multiplication operations between the corresponding WMs using Eqn. (6). We have designed and implemented a tiled matrix multiplication algorithm to carry out this step (see Figure 5). Our method is similar to the matrix multiplication with shared memory example in the CUDA SDK [24]. However, instead of dividing WMs into square matrices as in [24], we divide them into sub-matrices of size *Tile*_ *width *× *Bin*_ *number *in order to make full use of the power of shared memory. We call these sub-matrices the tiles. In Figure 5 each WM is divided into two tiles. Then the matrix multiplication *C *= *A *× *B *can be implemented as *C *= *Tile*1_*A *× *Tile*1_*B *+ *Tile*2_*A *× *Tile*2_*B*. Here, *C *is the joint WM. By dividing WMs into small tiles, we can store them in the fast shared memory and thus improve the performance greatly.

**Algorithm 5**: CUDA Kernel 4

   **Input**: WMs for each gene.

   **Output**: Pairwise MI values.

   **foreach ***i*, ≤ 1 ≤ *i *≤ *N ***- **1 **do**

      **foreach ***j*, *i *≤ *j *≤ *N ***do**

         Assign (*i*, *j*)-th pair of WM to one thread block;

         *R *× *R *threads in each thread block work in parallel following the two for-loops in Algorithm 2 to compute the joint WM by doing tiled matrix multiplication for the current WM pair;

         One thread in this block is used to compute the joint entropy and MI;

      **end**

   **end**

In practice, a cyclic procedure is used in the kernel program to implement the tiled matrix multiplications. Each thread block first loads the current tile pair from global memory to shared memory. Then each thread computes one element of the tiled multiplication matrix. In the subsequent iteration, the next tile pair is loaded and multiplied. The tiled multiplication matrix will then be updated. This procedure will continue until all tile pairs are computed. The final tiled multiplication matrix is the joint WM we want to compute. At last, we calculate the MI value for the current gene pair. Algorithm 5 shows the pseudocode of our CUDA implementation of the parallel algorithm for Kernel 4.

### Partitioning

**Algorithm 6**: CUDA-based MI estimation with partition

   **Input**: *N *genes, each with *M *experiments.

   **Output**: Pairwise MI values.

   /*Host program executed on CPU*/

   Initialize parameters controlling MI estimation;

   **Partition gene expression data into ***P ***groups **;

   **foreach ***i*, 1 ≤ *i *≤ *P ***do**

      /*Kernel program executed on each thread*/

      Load *i*-th gene data group into the GPU device memory and compute WM for each gene using Algorithm 1;

      Write the WMs to CPU RAM;

   **end**

   **Partition WMs into ***Q ***groups;**

   **foreach ***i*, 1 ≤ *i *≤ *Q ***do**

      /*Kernel program executed on each thread*/

      Load *i*-th WM group into the GPU device memory and compute MI values using Kernel 2, Kernel 3 and Kernel 4;

      Read MI values for current WM group back to CPU;

      Write MI values to les;

   **end**

The space complexity for storing all WMs is *O*(*N *× *M *× *R*). Using double precision floating point numbers this translates to 8 × *N *× *M *× *R *bytes of memory. The GPU global memory is not sufficient to load all WMs for parallel computation in the kernel for large datasets. Therefore, a method is required to partition the pairwise MI computation into a number of steps, where each step requires only a subset of WMs. By partitioning both the gene expression data and WMs into small groups, we can process large datasets using limited GPU device memory. Our partitioning method is illustrated in Algorithm 6.

The method shown in Algorithm 6 divides the gene expression data and WMs into smaller groups, which can be stored within the GPU global memory. Note that, the memory complexity of *O*(*N *× *M *× *R*) is dominated by the computational complexity of *O*(*N*^2 ^× *M *× *R*^2^). Therefore, the required data transfer time can be completely hidden by the computation time.

## Results and Discussion

We have implemented the double precision CUDA-MI using CUDA Toolkit 3.0 and evaluated it on the following CUDA-enabled hardware:

- Nvidia Tesla C2050: 1.15 GHz engine clock speed, 14 multiprocessors, 3 GB GDDR5 device memory, 48 KB shared memory/multiprocessor.

Tests have been conducted with this card installed in a PC with an Intel Quad-Core i7-920 2.66 GHz CPU, 12 GB RAM running Linux Fedora 10.

We have used CUDA-MI to estimate MI values for different gene expression datasets. Two types of datasets are used in our simulations. One type consists of real datasets, the other type contains randomly generated simulated datasets for testing scalability. The number of genes and experiments in these datasets are shown in Table 1. In this table, the "nne" and "nasc" prefixed datasets are downloaded from. The two Yeast datasets are downloaded from the Eisen Lab website http://rana.lbl.gov/EisenData.htm. They are all real gene expression datasets. To further test the scalability of our CUDA-MI, we have additionally used simulated datasets with a varying number of genes and experiments. The simulated datasets were produced by ourselves and are listed as *S*2000_1000 to *S*10000_4000 in Table 1. We have compared the performance of our implementation to a widely used B-spline function based sequential MI estimation program - MIBE which is available from the author of [7]. In our tests we have used the double precision and multi-threaded version of MIBE with four threads on our quad-core workstation. It is compiled by gcc 4.3.2 with all available compiler optimizations enabled and runs on an Intel Quad-Core i7-920 2.66 GHz CPU. In our experiments, we used the MIBE default parameters *R *= 10 and *k *= 3 for both MIBE and CUDA-MI. Both tools use double precision floating point accuracy.

**Table 1:** Datasets used for performance evaluation.

Dataset ID	Number of Genes	Number of Experiments
nne2048_911	2048	911

nne4096_911	4096	911

nasc2048_2996	2048	2996

nasc4096_2996	4096	2996

Yeast_6221_80	6221	80

Yeast_6307_215	6307	215

S2000_1000	2000	1000

S4000_1000	4000	1000

S8000_1000	8000	1000

S4000_3000	4000	3000

S4000_4000	4000	4000

S4000_5000	4000	5000

S10000_2000	10000	2000

S10000_3000	10000	3000

S10000_4000	10000	4000

Table 2 shows the runtime performance of MIBE and CUDA-MI for processing different gene expression datasets. From Table 2 we can see that CUDA-MI achieves speedups of up to 82 compared to the multi-threaded MIBE. Because of the big memory usages of datasets *S*10000_2000, *S*10000_3000, and *S*10000_4000, the partitioning method (discussed later in Methods) is used to process them. Performance using the partitioning method is shown in bold characters in Table 2. From Table 2 we can see that the speedup of CUDA-MI improves for a larger number of genes and measurements. There are two reasons for this observation. Firstly, there is higher arithmetic intensity for a larger number of genes and measurements. Secondly, the relative influence of the kernel overhead is reduced for bigger datasets. Table 3 shows the runtime performance of MIBE and CUDA-MI for processing the *nne*4096_911 dataset using different parameters. The value of spline order ranges from 3 to 5 and the number of bins ranges from 10 to 20. From Table 3 we can make two observations:

**Table 2:** Comparison of runtime (in seconds) between multi-threaded MIBE (4 threads) and CUDA-MI

ID	MIBE	CUDA-MI	Speedup
nne2048_911	220.87	14.78	14.9

nne4096_911	867.35	45.17	19.2

nasc2048_2996	2174.65	44.89	48.4

nasc4096_2996	8645.28	153.46	56.3

Yeast_6221_80	246.01	30	8.2

Yeast_6307_215	490.46	42.18	11.6

S2000_1000	229.52	14.58	15.7

S4000_1000	904.86	50.89	17.8

S8000_1000	3635.11	192.05	18.9

S4000_3000	5960.87	148.09	40.3

S4000_4000	7993.99	199.07	40.2

S4000 5000	13790.19	256.76	53.7

**S10000**_**2000**	**34315.69**	**520.12**	**66.0**

**S10000**_**3000**	**51705.18**	**781.73**	**66.1**

**S10000**_**4000**	**68799.74**	**838.74**	**82.0**

**Table 3:** Comparison of runtimes (in seconds) for processing the ***nne*4096_911 **dataset with various parameters.

Bin Number	Spline Order	MIBE (4 threads)	CUDA-MI	Speedup
10	3	867.35	45.17	19.2

10	4	867.24	46.18	18.8

10	5	868.3	46.54	18.7

15	3	1926.67	60.02	32.1

15	4	1947.45	60.83	32.0

15	5	1949.65	61.77	31.6

20	3	3468.07	104.45	33.2

20	4	3657.64	105.40	34.7

20	5	3658.83	105.61	34.6

1. The speedup does not change significantly with a larger value of spline order.

2. The speedup improves significantly with a larger number of bins.

The reason for Observation 1 is that the spline order *k *does not have much impact on the overall runtime of the MI estimation algorithm. On the contrary because of the quadratic item *R*^2 ^in the Big O notion, the value of *R *influences the performance greatly. From Algorithm 5 we can see that in CUDA-MI totally *R *× *R *threads are used. This means a larger number of threads are used with a larger number of bins. Therefore CUDA-MI can work more efficiently with larger number of bins *R*, which explains Observation 2. We also have compared the runtime performance of CUDA-MI to the MPI based TINGe software [23] running with MPI installed on an Intel Quad-Core i7-920 and eight MPI processes for the datasets shown in Table 1. For all tested datasets except the *nasc*2048_2996 dataset, MIBE is able to outperform TINGe on the same hardware. Thus, we have decided only to include a runtime comparison between CUDA-MI and MIBE in this paper.

**Figure 6:**
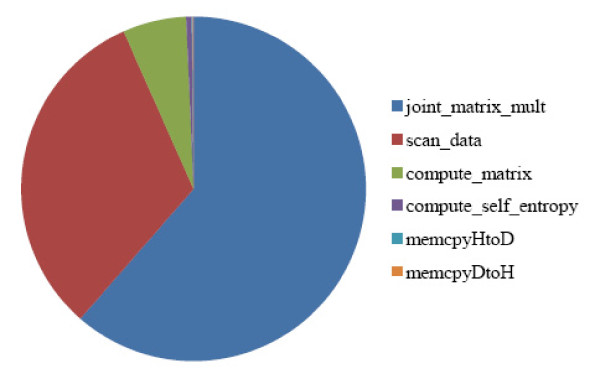
**Pie chart of CUDA-MI kernel functions using the visual profiler for dataset *nne*4096_911**.

Figure 6 shows the CUDA-MI runtime pro le for dataset *nne*4096_911(created using the CUDA profiler). It can be seen that the Kernel 4 ("joint_matrix_mult") takes 61.39% of the total runtime. Kernel 2 ("scan_data") occupies 31:93% of the total runtime. As Kernel 2 is optional, the runtime for the CUDA-MI can be further reduced if Kernel 2 is bypassed.

Further profiling of CUDA-MI is performed with fixed spline order 3 and different bin numbers, 10, 15, 20. The bin number determines the number of threads used in Kernel 4 and therefore influences efficiency and complexity. The results in Table 4 show that the GPU runtime for Kernel 4 takes 61.39%, 71.9%, 79.38% of the total runtime for different bin number. The amount of floating point operations for the matrix multiplication in Kernel 4 is *N*^2 ^× *M *× *R*^2^. The GFlops for matrix multiplication in Kernel 4 can then be computed as *N*^2 ^× *M *× *R*^2^/*t *where *t *is the Kernel 4 runtime in Table 4. It can be seen that the GFlops for matrix multiplication is increased by using a larger bin number. This is because CUDA-MI works more efficiently with larger number of bins.

**Table 4:** GFlops and GPU runtime (in Sec.) of Kernel 4 for dataset ***nne*4096_911 **with fixed spline order and variable bin number.

Bin Number	Runtime	Percentage	GFlops
10	13.24	61.39%	115.41

15	22.96	71.9%	149.74

20	36.93	79.38%	165.56

We have used the SynTReN [25] software to generate synthetic datasets from known underlying gene networks. Three synthetic networks are generated as shown in Table 5. The networks consist of the same number of genes (250) and a variable number of experiments (500, 900, and 1200). In order to use CUDA-MI to infer GRNs, we convert its output into an adjacency matrix. The adjacency matrix is then used by ARACNE [14] to infer GRNs. We call this method "C-ARACNE". We have used the same parameters for ARACNE and TINGe for inferring GRNs, i.e., the P-value for MI threshold is 0.00001 and DPI tolerance is 0.01. TINGe runs on 4 cores using 8 MPI processes. As the underlying network structure is known, we can compute the true positive (TP), true negative (TN), false positive (FP), and false negative (FN) by comparing the output with the known network. In this paper, TP stands for the correctly inferred edges, TN stands for the correctly removed edges, FP represents wrongly added edges, and FN refers to wrongly removed edges. We have calculated sensitivity, specificity and precision as follows: *specificity *, *sensitivity *, and *precision *.

**Table 5:** Comparison of ARACNE, TINGe and C-ARACNE using synthetic networks.

		Time (Sec.)	Specificity	Sensitivity	Precision	TP	TN	FP	FN
m = 500	ARACNE	188.28	0.995	0.255	0.373	98	30575	165	287
	
	TINGe	3.74	0.995	0.257	0.404	99	30594	146	286
	
	C-ARACNE	1.07	0.996	0.291	0.448	112	30602	138	273

m = 900	ARACNE	637.56	0.994	0.27	0.378	104	30569	171	281
	
	TINGe	3.9	0.995	0.265	0.394	102	30583	157	283
	
	C-ARACNE	1.3	0.996	0.294	0.454	113	30604	136	272

m = 1200	ARACNE	1312.3	0.995	0.283	0.394	109	30572	168	276
	
	TINGe	4.86	0.995	0.273	0.4	105	30581	159	280
	
	C-ARACNE	1.46	0.995	0.296	0.44	114	30595	145	271

The results for inferring GRNs using ARACNE, TINGe, and C-ARACNE are shown in Table 5. From Table 5 we can see that C-ARACNE achieves the best performance in terms of both runtime and quality of inferred GRNs.

## Conclusions

In this paper we have proposed a CUDA-based parallel algorithm - CUDA-MI for accelerating MI estimation using the B-spline function. In order to exploit the GPU's capabilities to accelerate MI estimation, we have used the fast shared memory, fine-grained parallelism, and partitioning to implement our algorithm. Our implementation achieves speedups up to 82 compared to the multi-threaded MIBE on a modern Intel quad-core. This result indicates that CUDA-enabled architectures are a highly efficient hardware platform for this type computation. We also have used the output of CUDA-MI to infer GRNs. Our experiments show that compared to ARACNE and TINGe, CUDA-MI can achieve better performance in terms of both runtime and inferred GRNs' quality for synthetic datasets.

## Availability and requirements

• Project name: CUDA-MI

• Project home page: https://sites.google.com/site/liuweiguohome/cuda-mi

• Operating System: Linux

• Programming language: CUDA and C

• Other requirements: CUDA SDK and Toolkits 3.0 or higher, CUDA-enabled GPU with at least 3 G memory.

• License: none

## Competing interests

The authors declare that they have no competing interests.

## Authors' contributions

HS conceptualized the study, carried out the design and implementation of the algorithm, performed benchmark tests, analyzed the results and drafted the manuscript; BS and WL conceptualized the study, participated in the algorithm optimization and analysis of the results and contributed to the revising of the manuscript; WMW conceptualized the study and contributed to the revising of the manuscript. All authors read and approved the final manuscript.
